# Severity of SARS-CoV-2 Omicron BA.2 infection in unvaccinated hospitalized children: comparison to influenza and parainfluenza infections

**DOI:** 10.1080/22221751.2022.2093135

**Published:** 2022-07-04

**Authors:** Winnie W.Y. Tso, Mike Y.W. Kwan, Yu Liang Wang, Lok Kan Leung, Daniel Leung, Gilbert T. Chua, Patrick Ip, Daniel Y.T. Fong, Wilfred H.S. Wong, Sophelia H.S. Chan, Jasper F.W. Chan, Malik Peiris, Yu Lung Lau, Jaime S. Rosa Duque

**Affiliations:** a Department of Paediatrics and Adolescent Medicine, The University of Hong Kong, Hong Kong, People’s Republic of China; b State Key Laboratory of Brain and Cognitive Sciences, The University of Hong Kong, Hong Kong, People’s Republic of China; c Department of Paediatrics and Adolescent Medicine, Princess Margaret Hospital, Hong Kong, People’s Republic of China; d School of Nursing, The University of Hong Kong, Hong Kong, People’s Republic of China; e State Key Laboratory of Emerging Infectious Diseases, Carol Yu Centre for Infection, Department of Microbiology, The University of Hong Kong, Hong Kong, People’s Republic of China; f School of Public Health, The University of Hong Kong, Hong Kong, People’s Republic of China

**Keywords:** Covid-19, omicron, neurological, respiratory, children

## Abstract

There has been a rapid surge of hospitalization due to the severe acute respiratory syndrome coronavirus 2 (SARS-CoV-2) Omicron variants globally. The severity of Omicron BA.2 in unexposed, unvaccinated, hospitalized children is unknown. We investigated the severity and clinical outcomes of COVID-19 infection during the Omicron wave in uninfected, unvaccinated hospitalized children and in comparison with influenza and parainfluenza viral infections. This population-based study retrieved data from the HK territory-wide CDARS database of hospitalisations in all public hospitals and compared severe outcomes for the Omicron BA.2-dominant fifth wave (5–28 February 2022, n = 1144), and influenza and parainfluenza viruses (1 January 2015–31 December 2019, n = 32212 and n = 16423, respectively) in children 0–11 years old. Two deaths (0.2%) out of 1144 cases during the initial Omicron wave were recorded. Twenty-one (1.8%) required PICU admission, and the relative risk was higher for Omicron than influenza virus (n = 254, 0.8%, adjusted RR = 2.1, 95%CI 1.3–3.3, *p* = 0.001). The proportion with neurological complications was 15.0% (n = 171) for Omicron, which was higher than influenza and parainfluenza viruses (n = 2707, 8.4%, adjusted RR = 1.6, 95%CI 1.4–1.9 and n = 1258, 7.7%, adjusted RR = 1.9, 95%CI 1.6–2.2, *p* < 0.001 for both, respectively). Croup occurred for Omicron (n = 61, 5.3%) more than influenza virus (n = 601, 1.9%, adjusted RR = 2.0, 95%CI 1.5–2.6, *p* < 0.001) but not parainfluenza virus (n = 889, 5.4%). Our findings showed that for hospitalized children who had no past COVID-19 or vaccination, Omicron BA.2 was not mild. Omicron BA.2 appeared to be more neuropathogenic than influenza and parainfluenza viruses. It targeted the upper airways more than influenza virus.

## Introduction

During the pre-vaccination era of Coronavirus disease 2019 (COVID-19), 2,128,587 of 72.8 million children in the United States (US) were infected by the wild-type SARS-CoV-2 in the first year of the pandemic[1,2]. Since then, other variants, such as Alpha, Beta, Gamma and Delta also swept across the globe[3–12] Many who survived developed immunity across SARS-CoV-2 strains[13–15]. Therefore, the severity of Omicron in hospitalized children previously unexposed to COVID-19 is unknown[16,17].

The setting in Hong Kong (HK) can provide a prototypical platform for understanding the severity of Omicron in children. Since the onset of the pandemic, the HK Government implemented stringent social distancing policies, including universal masking, contact tracing, intermittent business closures and territory-wide school suspensions[18–22]. These measures were associated with lowest numbers of COVID-19 (an infection rate of ∼1% of the population of HK as of 31 October 2021) and near disappearance of influenza and parainfluenza viral infections in HK[23,24]. Furthermore, COVID-19 vaccines were only approved for HK children aged 5–11 years old in mid-January 2022, so these children remained both uninfected and unvaccinated by the start of the Omicron wave[25]. The emergence of Omicron, predominantly BA.2 in HK, led to exponential increases of SARS-CoV-2 infections during the fifth wave of COVID-19[23,26]. Indeed, although more children than older adults had experienced favorable outcomes when infected by pre-Omicron SARS-CoV-2, recent studies suggested disproportionately higher hospitalization rates in children after the emergence of Omicron[27,28]. Furthermore, these cases had a greater predilection for more severe complications affecting the neurological and respiratory systems[15,29–32]. In an observational study of the first Omicron wave, likely predominated by the BA.1 sublineage, the most frequent clinical diagnoses linked to pediatric hospitalization was seizure[15]. Other studies suggested that laryngotracheobronchitis, or croup, is more prevalent, severe and prolonged with Omicron than other variants[30–32]. Deaths in children infected with Omicron have occurred but were related to complex underlying co-pathologies[15]. Such emerging data are reshaping the notion that Omicron is possibly not as mild as initially speculated[33].

This population-based study aimed to describe Omicron BA.2-dominant fifth wave’s severe clinical outcomes that included mortality, pediatric intensive care unit (PICU) admissions, neurological and respiratory complications in hospitalized children aged 0–11 years, who basically lack immune exposure to past COVID-19 infections or vaccination. Additionally, we compared these severe complication outcomes in the hospitalized children of this age group during this fifth wave of COVID-19 to influenza and parainfluenza viruses from 2015–2019, which commonly caused seizures and croup, respectively[24].

## Methods

### Study design and patient groups

We conducted a population-based cohort study of hospitalized children 0–11 years old in HK by analyzing electronic medical records retrieved from the Clinical Data Analysis and Reporting System (CDARS). CDARS is an HK territory-wide health registration system recording admissions of all 42 public hospitals. The database has been used for many high-quality, population-based studies with accurate coding[34]. CDARS captures essentially all patient diagnoses the 5 years before and during the COVID-19 pandemic.

During the first 4 waves of the COVID-19 pandemic in HK that predated the Omicron, almost all infected children, regardless of disease severity, were admitted to public hospitals according to the government’s isolation policy[35]. However, during the Omicron wave, due to the overwhelming number of infected cases, children with moderate to severe diseases were admitted to public hospitals for disease management while others remained home. Most children infected with influenza or parainfluenza viruses with moderate to severe diseases before the COVID-19 pandemic, especially those requiring pediatric intensive care unit (PICU), were also admitted to public hospitals, which had a similar practice as the Omicron wave.

This study describes the clinical severity of Omicron in hospitalized children. Additionally, severe outcomes that included neurological and respiratory complications of hospitalized children with COVID-19 during the Omicron wave were compared with those who had influenza and parainfluenza viral infections. Due to differences in the triage criteria for hospital admission during the first 4 waves and the low sample size, outcomes during the first 4 waves (between 1 January 2020 and 1 November 2021) are shown as separate supplementary files only (Supplementary Table 5).

Data were retrieved from CDARS using ICD-9 diagnostic codes (Supplementary Table 1). The study period for the Omicron fifth wave was 5 February to 28 February 2022. Mortality information published by the Centre for Health Protection (CHP) of the HK Government was counterchecked for reference only, which were not included in the final analyses[36]. Hospitalized influenza and parainfluenza viral infections between 1 January 2015 and 31 December 2019 were obtained.

The inputted ICD-9 coding was based on diagnoses of infections by the specific viruses according to symptomatology and laboratory confirmation using the immunofluorescence assay and/or reverse transcription-polymerase chain reaction (RT–PCR) on their respiratory tract specimens. Similarly, children were diagnosed with COVID-19 using RT–PCR on their respiratory tract specimens. Children with the principal diagnoses of COVID-19 or COVID-19-related symptoms/conditions, e.g. seizures, croup, upper respiratory tract infection, gastroenteritis, etc., with a co-diagnosis of COVID-19 or chronic diseases, e.g. cancers, chronic kidney diseases, etc., who were particularly prone to have COVID-19-related illnesses, were regarded as admission due to COVID-19 infection. Cases with co-infections of 2 or more respiratory viruses/SARS-CoV-2 were excluded.

We extracted and categorized data according to the following outcomes:

- Mortality and severe complications:
  1. Number of death cases
  2. Pediatric intensive care (PICU) admissions
  3. Mechanical ventilation
  4. Oxygen use
- Neurological complications:
  1. Seizures
    - Benign febrile seizures: non-focal seizures and fever on patients between 6 months to 5 years old, with no known history of epilepsy,
    - Seizure with fever: seizure association with fever on patients who are < 6 months and ≥6 years old, with no known history of epilepsy,
    - Epilepsy with breakthrough seizures: Known history of epilepsy with recurrence of seizure during the febrile illness
  2. Encephalitis/encephalopathy
- Respiratory complications:
  1. Croup
  2. Pneumonia

To optimize the accuracy of the indication for PICU admission, only cases with diagnostic or procedural coding consistent with actual clinical needs for intensive/critical care were counted. Additional data including the child’s demographic details were extracted for statistical analyses.

This study was approved by the University of Hong Kong/Hospital Authority Hong Kong West Cluster (HKU/HKW IRB UW 22-204) and the Kowloon West Cluster Research Ethics Committee Institutional Review Boards (KWC-REC KW/FR-20-086 (148-10)).

### Statistical analysis

To compare the specified outcomes (dependent variables) between hospitalized cases with Omicron BA.2, influenza and parainfluenza infections (independent variables), a quasi-Poisson regression was conducted to estimate the corresponding relative risks (RRs). Both the crude and adjusted estimates for age, sex and comorbidities (potential confounding independent variables) were obtained. We selected co-morbid conditions that were relevant to this age group according to the pediatric co-morbidity index[37]. To further assess the potential for seasonality influences, we repeated the quasi-Poisson regression on the subgroup of influenza seasons, January-April and July-August, which detected no difference between the main analysis and the subgroup analysis accounting for seasonality (Supplementary Table 4)[38]. To explore the potential influence of age, subgroup analyses were performed for children ages between 0–5 years and 6–11 years (Supplementary Tables 2a, 2b and 3), and their differences were assessed by age interaction. All estimates were accompanied by 95% confidence intervals (CIs), and the statistical tests used a 5% nominal level of significance. The statistical analyses were conducted in R 4.1.3 (R Core Team, Vienna, Austria.).

## Results

### Hospitalization due to covid-19 and other viral infections

1144 children aged 0–11 years were hospitalized during the Omicron wave from 5 February to 28 February 2022 (Table 1). There were 918 (80.2%) aged 0–5 years (Supplementary Tables 2a and 2b). For all other viruses, 32,212 children were hospitalized due to influenza (75.5% aged 0–5 years) and 16423 children due to parainfluenza viral infections (93.0% aged 0–5 years).

**Table 1:** . Clinical characteristics of all hospital admissions between Omicron BA.2 and influenza and parainfluenza viral infections.

	SARS-CoV-2: Omicron BA.2 (n = 1144)	Influenza (n = 32212)	*p*-value^+^ (Omicron vs Influenza)	Parainfluenza (n = 16423)	*p*-value^+^(Omicron vs Parainfluenza)
Data period, day/month/year	05/02/2022–28/02/2022	01/01/2015–31/12/2019		01/01/2015–31/12/2019	
Sex					
Male	658 (57.5%)	17504 (54.3%)	0.04*	9228 (56.2%)	0.39
Female	486 (42.9%)	14708 (45.7%)		7159 (43.8%)	
Age mean in years (SD)	3.4 (3.1)	4.1 (2.8)	<0.001***	2.6 (2.1)	<0.001***
0–5 years old	918 (80.2%)	24334 (75.5%)		15268 (93.0%)	
6–11 years old	226 (19.8%)	7878 (24.5%)		1155 (7.0%)	
Comorbidities	71 (6.2%)	1592 (4.9%)	0.06	1393 (8.5%)	0.008**
Mortality and severe complications					
Death cases	2 (0.2%)^‡^	16 (0.1%)		7 (0.04%)	
PICU admissions	21 (1.8%)	254 (0.8%)		270 (1.6%)	
Mechanical ventilation	8 (0.7%)	82 (0.3%)		106 (0.7%)	
Oxygen use	11 (1.0%)	120 (0.4%)		225 (1.4%)	
Neurological complications	171 (15.0%)	2707 (8.4%)		1258 (7.7%)	
All seizures	166 (14.5%)	2650 (8.2%)		1248 (7.6%)	
Febrile seizures	133 (11.6%)	2303 (7.2%)		1142 (7.0%)	
Seizures with fever	28 (2.5%)	290 (1.0%)		42 (0.3%)	
Breakthrough seizures with epilepsy	5 (0.4%)	57 (0.2%)		64 (0.4%)	
Encephalitis/encephalopathy	5 (0.4%)	78 (0.2%)		17 (0.1%)	
Respiratory complications	70 (6.1%)	2343 (7.3%)		2891 (17.6%)	
Croup	61 (5.3%)	601 (1.9%)		889 (5.4%)	
Pneumonia	10 (0.9%)	1756 (5.5%)		2030 (12.4%)	
Croup/pneumonia ratio	6.10	0.34		0.44	

Data are n (%) unless otherwise specified. PICU = paediatric intensive care units.

*p < 0.05,

**p < 0.01,

***p < 0.001.

^+^ Fisher's Exact test was used when comparing binary variables, independent t-test was used when comparing two continuous variables.

^‡^ Two deaths were extracted from CDARS. During this study period, 4 total deaths were recorded by the Centre for Health Protection, Hong Kong, since these 2 deaths occurred in the Accident & Emergency Department and therefore were not inputted into CDARS by the admitted in-patient wards.

### Mortality due to covid-19 and other viruses

Amongst the 1144 Omicron cases, 2 COVID-19-associated deaths were identified (Table 1). In addition, CHP reported 2 other deaths during the study period. They were 11 months, 3, 4 and 9 years old. Three had good past health. The 9-year-old child had Duchenne muscular dystrophy. None were vaccinated against COVID-19. The cause of death for 2 cases was neurological: one with encephalopathy and the other with fulminant cerebral oedema, which recently became a recognized phenotype of encephalitis^27^. Only 2 deaths due to Omicron were used for comparison with other viruses so that all information between groups were equally obtained from CDARS. The number of death due to Omicron was 2 out of 1144 (0.2%), and the adjusted relative risk of death was higher than parainfluenza virus (7 out of 16423, 0.04%; adjusted RR = 4.7, [95% CI (1.1–19.6)], *p* = 0.035) but not influenza virus (16 out of 32,212, 0.1%; adjusted RR = 2.7, [95% CI 0.5–15.7], *p* = 0.257) (Table 2).

**Table 2:** . Relative risks of complications for Omicron BA.2 in comparison to influenza and parainfluenza viral infections.

	SARS-CoV-2 Omicron BA.2 vs Influenza				SARS-CoV-2 Omicron BA.2 vs Parainfluenza			
	Crude		Adjusted^+^		Crude		Adjusted^+^	
	RR (95% CI)	*p*-value	RR (95% CI)	*p*-value	RR (95% CI)	*p*-value	RR (95% CI)	*p*-value
Severe complications								
Death cases	3.5 (0.8–15.3)	0.09	2.7 (0.5–15.7)	0.26	4.1 (0.9–19.7)	0.08	4.7 (1.1–19.6)	0.04*
PICU admissions	2.3 (1.5–3.6)	<0.001***	2.1 (1.3–3.3)	0.001**	1.1 (0.7–1.7)	0.62	1.2 (0.8–1.9)	0.37
Mechanical ventilation	2.7 (1.3–5.7)	0.006**	2.3 (1.1–4.9)	0.03*	1.1 (0.5–2.2)	0.83	1.3 (0.6–2.6)	0.55
Oxygen use	2.6 (1.4–4.8)	0.003**	2.3 (1.2–4.2)	0.009**	0.7 (0.4–1.3)	0.25	0.8 (0.4–1.4)	0.40
Neurological complications	1.8 (1.5–2.1)	<0.001***	1.6 (1.4–1.9)	<0.001***	2.0 (1.7–2.3)	<0.001***	1.9 (1.6–2.2)	<0.001***
All seizures	1.8 (1.5–2.0)	<0.001***	1.6 (1.4–1.9)	<0.001***	1.9 (1.6–2.2)	<0.001***	1.9 (1.6–2.2)	<0.001***
Febrile seizures	1.6 (1.4–1.9)	<0.001***	1.4 (1.2–1.6)	<0.001***	1.7 (1.4–2.0)	<0.001***	1.7 (1.4–2.0)	<0.001***
Seizures with fever	2.7 (1.8–4.0)	<0.001***	3.0 (2.1–4.2)	<0.001***	9.6 (5.9–15.4)	<0.001***	4.3 (2.4–7.6)	<0.001***
Breakthrough seizures with epilepsy	2.5 (1–6.2.0)	0.05	NA	NA	1.1 (0.5–2.8)	0.81	NA	NA
Encephalitis/ encephalopathy	1.8 (0.7–4.5)	0.20	1.8 (0.8–4.2)	0.17	4.2 (1.6–11.4)	0.005**	5.3 (1.6–17.9)	0.007**
Respiratory complications	0.8 (0.7–1.1)	0.14	0.8 (0.7–1.0)	0.09	0.3 (0.3–0.4)	<0.001***	0.3 (0.3–0.4)	<0.001***
Croup	2.9 (2.2–3.7)	<0.001***	2.0 (1.5–2.6)	<0.001***	1.0 (0.8–1.3)	0.91	1.0 (0.8–1.3)	0.74
Pneumonia	0.2 (0.1–0.3)	<0.001***	0.2 (0.1–0.3)	<0.001***	0.1 (0.03–0.1)	<0.001***	0.1 (0.03–0.1)	<0.001***

RR = relative risk, CI = confidence interval.

^+^ Model was adjusted by age, sex and comorbidities.

*p < 0.05,

**p < 0.01,

***p < 0.001.

NA = not applicable as epilepsy is a co-morbid condition

### PICU admission due to covid-19 and other viral infections

Twenty-one (1.8%) children with COVID-19 infection required PICU admission during the Omicron wave (Table 1). 254 (0.8%) children with influenza and 270 (1.6%) children with parainfluenza viruses required PICU admission over the 5-year study period. The adjusted RRs of PICU admission for Omicron were higher than influenza virus (adjusted RR = 2.1, [95% CI 1.3–3.3], *p* = 0.001) but similar to parainfluenza virus (Table 2). The risk of Omicron for PICU admission compared to influenza virus in children aged 6–11 years was higher than the younger age group (Supplementary Table 3). Eight (0.7%) children with Omicron required mechanical ventilation, which was higher than influenza virus (n = 82, 0.3%, adjusted RR = 2.3, [95% CI 1.1–4.9], *p* = 0.03) and similar to parainfluenza virus (n = 106, 0.7%). Eleven (1.0%) had oxygen use, which was higher than influenza virus (n = 120, 0.4%, adjusted RR = 2.3, [95% CI 1.2–4.2], *p* = 0.009) but not parainfluenza virus (n = 225, 1.4%).

### Neurological complications due to covid-19 and other viruses

171 (15.0%) children with neurological complications were hospitalised during the Omicron-dominant wave (Table 1). The most common neurological complication was febrile seizure (n = 133, 11.6%), followed by seizure with fever (n = 28, 2.5%). Five (0.4%) had COVID-associated encephalitis/encephalopathy.

The adjusted relative risk of all seizure types in children with Omicron was higher than children with influenza (n = 2650, 8.2%, adjusted RR = 1.6, [95% CI 1.4–1.9], *p* < 0.001) and parainfluenza viruses (n = 1248, 7.6%, adjusted RR = 1.9, [95% CI 1.6–2.2], *p* < 0.001). The seizure RR of Omicron compared to influenza or parainfluenza viruses was higher in children aged 6–11 years than the younger age group (*p* = 0.37 and *p* = 0.042 respectively) (Supplementary Table 3). There were more febrile seizures among Omicron infected children (n = 133, 11.6%) than influenza (n = 2303, 7.2%, adjusted RR 1.4, [95% CI 1.2–1.6], *p* < 0.001) or parainfluenza viruses (n =  1142, 7.0% adjusted RR 1.7, [95% CI 1.4–2.0], *p* < 0.001). Children with Omicron had a increased risk of seizure with fever (n = 28, 2.5%) compared to influenza (n = 290, 1.0%, adjusted RR = 3.0, [95% CI 2.1–4.2], *p* < 0.001) or parainfluenza (n = 42, 0.3%, adjusted RR = 4.3, [95% CI 2.4–7.6], *p* < 0.001) viruses (Table 2). Children with Omicron had a increased risk of encephalitis/encephalopathy (n = 5, 0.4%) compared to parainfluenza virus (n = 17, 0.1%, adjusted RR = 5.3, [95% CI 1.6–17.9], *p* = 0.007), but not influenza virus (n = 78, 0.2%).

### Respiratory complications due to covid-19 and other viruses

Sixty-one (5.3%) with Omicron developed croup, which was higher than influenza (n = 601, 1.9%, adjusted RR = 2.0, [95% CI 1.5–2.6], *p* < 0.001), but not parainfluenza (n = 889, 5.4%) viruses (Tables 1 and 2). The proportions of pneumonia during the Omicron-dominant wave (n = 10, 0.9%) were lower than either influenza or parainfluenza viruses (n = 1756, 5.5%, adjusted RR = 0.2, 95% CI 0.1–0.3 and n = 2030, 12.4%, adjusted RR = 0.1, 95% CI 0.03–0.1, *p* < 0.001 for both, respectively) (Table 2).

## Discussion

This is the first population-based cohort study of hospitalized children that describes the severe clinical manifestations of Omicron BA.2. Additionally, this study found that severe outcomes due to Omicron BA.2 were not less than influenza and parainfluenza viruses. There was a higher risk of death from Omicron BA.2 than parainfluenza virus, and Omicron BA.2 was more pathogenic than influenza virus, resulting in more PICU admissions, mechanical ventilation and oxygen use. Omicron BA.2 was neuropathogenic, as patients had more seizures than influenza and parainfluenza infections. Importantly, the risks of encephalitis/encephalopathy were higher for Omicron BA.2 than parainfluenza virus. Omicron BA.2 caused more disease in the upper airway compared to influenza virus but less pneumonia.

Our study confirms previous findings from South Africa that the Omicron variant might be associated with increased seizure risks^8^. Prior to the Omicron outbreak, seizure was not a common manifestation of pediatric COVID-19[38,39]. Since the Omicron surge, 15.0% of hospitalized children with COVID-19 were admitted due to neurological complications compared to the 3.8% reported for other SARS-CoV-2 variants during the pre-vaccinated era in the UK[40]. A majority (80%) were simple febrile seizure, but 17% of seizures were out of its typical diagnostic age range. The risks of seizure for Omicron BA.2 from this study was 1.6–1.9 more than influenza and parainfluenza viruses. The risks of seizure with fever in children aged ≥6 years were even higher, at 3–4 times higher than influenza and parainfluenza viruses. In view of the 2 deaths related to COVID-associated encephalitis/encephalopathy, it will be important for pediatricians to anticipate such complication, in particular, how to differentiate acute encephalitis from febrile seizure and initiate medical interventions promptly.

Recently, croup due to Omicron has been described to be more severe and persistent[30,32]. Omicron had replaced parainfluenza as the predominant cause of viral croup during the initial phase of the Omicron variant surge according to a small study in the US, where the incidence of croup due to Omicron had doubled[30,31]. These patients were more likely to require nebulized epinephrine[30]. In HK, there was a >2-fold increase in croup for Omicron BA.2 than influenza, while similar to parainfluenza. This observation that Omicron preferentially affects the upper airway is supported by *ex vivo* human lung cultures demonstrating Omicron replicates better in the bronchus than lungs[41].

Our finding that Omicron BA.2 is associated with severe hospital outcomes in children was consistent with a recent age-specific analysis of hazard ratio (HR) of hospital admissions in England with Omicron BA.1 compared with Delta[42]. This national cohort study found considerable variation in the severity of Omicron BA.1 relative to Delta cases with age. The adjusted HRs for hospital admission did not differ between these 2 variants for children <10 years old (HR 1.1), while this was greatly reduced for adults aged 20–69 years old (HR 0.25). This analysis was adjusted for multiple confounders, so that the intrinsic severity of Omicron could be assessed relative to that of Delta in different age groups. In a secondary analysis disaggregating further those younger than 10 years old, the relative risk was higher in those younger than 1 year of age than in those aged 1–4 years or 5–9 years. In parallel, the 2 in-hospital deaths from our study were <5 years old.

Possible explanations as to why hospitalized children with Omicron BA.2 in HK have such clinical complications could be related to their low exposure to previous COVID-19 variants due to the strict social-distancing practices[5]. The effectiveness of such measures is substantiated by the near-complete disappearance of some communicable diseases, including influenza and parainfluenza viruses, in the HK community since early 2020[24]. Furthermore, the vaccination rate, especially in young children, was extremely low at the beginning of the Omicron surge. As of 12 February 2022, only 6% of children age 5–11 years had received their 1st dose of vaccination[25]. Therefore, it is possible that unexposed, unvaccinated, hospitalized children are more vulnerable due to their lack of cross-reactive immunity, in particular, the more durable and broader T cell immunity[43–45].

This study had limitations. First, genomic sequencing information is not encoded in CDARS, so not all included COVID-19 cases were caused by Omicron BA.2. However, based on epidemiological data, even at the start of the fifth wave, Omicron had become the dominant strain, accounting for >60% of the total cases[46]. CHP reported that out of the 130 COVID-associated deaths 3 February to 2 March 2022 with available genome sequencing results, 117 were Omicron and only 13 were Delta[47]. Since epidemiological criteria to describe the type of SARS-CoV-2 infection do not provide certainty on Omicron BA.2, future research can be enriched by completion of genomic sequencing results for all participants. Second, we caution our in-hospital mortality of 0.2%, which does not reflect the overall deaths in the community because some children with mild COVID-19 during the Omicron wave were not admitted, and CHP reported 0.02% case-fatality rate (CFR) for 0–9 years old children at the time of this writing on 15 March 2022[48]. However, this information emphasizes that clinicians should anticipate potential deterioration for hospitalized pediatric patients as they are at risk of severe outcomes. Third, the comparison of Omicron with influenza and parainfluenza viruses were made from disjoint time periods. During January 2020 to February 2022, almost all children with COVID-19 were hospitalized in public hospitals and facilities only, whereas for children infected with influenza or parainfluenza viruses, they had the option of in-hospital care in the public or the private sector. Nevertheless, based on our clinical experience, there were no major calendar-period-specific changes in nasopharyngeal testing practices or overall healthcare-seeking behaviors of HK parents when their child became acutely ill. Furthermore, official statistics showed CDARS accounted for 85% of all hospitalization in HK[49]. Even in the pre-COVID-era, almost all children with serious neurological or respiratory compromise were transferred to public hospitals due to the lack of pediatric intensive care support in the private sector. These factors limit potential underestimation of severe influenza and parainfluenza-related complications. Fourth, we were unable to perform extensive analyses for the first 4 waves of COVID-19 because of its low sample size in HK even as CDARS should have captured essentially all these cases that required admission to public hospitals under HK Government’s isolation policies. Finally, information on the influenza virus infection by type/subtypes or vaccination status was unavailable for all individual patients in this study, precluding data analyses on the effects of these potential factors. Recent publications from our group and CHP demonstrated that during the study period of 2015–2019, the predominant influenza viruses in HK were H1N1, H3N2 and B in HK, which varied by month[38,50,51]. Moreover, although the HK community of children had 12% influenza vaccine uptake in 2015 to recently up to 69.2% for those attending primary schools enrolled in the vaccination scheme that began in 2018, the vaccination rates had been merely 8–12% for children who were hospitalised[38,50–52]. For clearer delineation between the severity of the 2 viruses, further research specifically comparing COVID-19 with those who had been uninfected and unvaccinated with the influenza virus only will be required. The strengths of this study included the large sample size of the study design that allowed for controlling confounders such as age, sex, co-morbidities and influenza seasonality. Additionally, these study data were reviewed by bioinformatics statisticians, physicians and a virologist with a spectrum of subspecialty expertise to assure relevant clinical implication of disease complications.

In conclusion, Omicron BA.2 can cause severe disease in unvaccinated, hospitalized children who had low exposure to coronaviruses the past 2 years. As many studies have demonstrated COVID-19 vaccination efficacy in reducing severe complications and deaths[53], it is necessary to advocate for immunization access, particularly for vulnerable children as recommended by major health organizations, to minimize vaccine-preventable diseases[54–60].

## Supplementary Material

Supplemental Material

## Data Sharing

All data used in this analysis were anonymised. Raw data can be obtained from JS Rosa Duque, and request for access to the underlying CDARS source information can be directed to HK Hospital Authority. Data from HK Centre for Health Protection were publicly available. The R script for performing the main statistical analysis of this paper is shown in the Supplementary Appendix.
