# Supplementary material for: Severity of SARS-CoV-2 Omicron BA.2 infection in unvaccinated hospitalized children: comparison to influenza and parainfluenza infections
Source: Emerg Microbes Infect. 2022 Jul 4;11(1):1742–50. doi: 10.1080/22221751.2022.2093135 (PMC9258055; doi:10.1080/22221751.2022.2093135)
Supplement: Supplemental Material [file TEMI_A_2093135_SM9542.zip › V8 Table S1.docx]

***Supplementary Table 1:* List of International Classification of Diseases (ICD-9) codes corresponding to the viruses, associated complications and comorbidities.**

| **Disease** | **ICD-9 code** |
| --- | --- |
| COVID-19 | 519.0 (8) |
| Influenza | 487.0, 487.1, 487.8 |
| Parainfluenza | 079.89 (4), 460.0 (8), 480.2 |
|  |  |
| **Complications** |  |
| Febrile seizures/seizures with fever | 780.31, 780.32 |
| Encephalitis/encephalopathy | 323.9, 348.3 |
| Croup | 464.4 |
| Pneumonia | 486.0 |
|  |  |
| **Comorbidities** |  |
| Anaemia | 280, 281, 282, 283, 284, 285 |
| Anxiety | 300 |
| Any malignancy | 140-239 |
| Asthma | 493 |
| Cardiovascular conditions | 390-459 |
| Chromosomal anomalies | 758 |
| Congenital anomalies | 740-757, 759 |
| Depression | 311 |
| Developmental delays | 315 |
| Diabetes mellitus | 250 |
| Epilepsy | 345 |
| Gastrointestinal conditions | 530-539, 570-579, 555-558, 560, 567-569 |
| Pains | 338 |
| Sleep disorders | 307.4, 347 |
| Weight loss | 783.21 |
| Conduct disorder | 312 |
| Joint disorder | 718, 719 |
| Nausea and vomiting | 787.01 |
