# Supplementary material for: Severity of SARS-CoV-2 Omicron BA.2 infection in unvaccinated hospitalized children: comparison to influenza and parainfluenza infections
Source: Emerg Microbes Infect. 2022 Jul 4;11(1):1742–50. doi: 10.1080/22221751.2022.2093135 (PMC9258055; doi:10.1080/22221751.2022.2093135)
Supplement: Supplemental Material [file TEMI_A_2093135_SM9542.zip › V8 Table S2.docx]

***Supplementary Table 2a:* Complications among the 3 viral infections between age 0 to 5 years.**

|  | **SARS-CoV-2: Omicron BA.2**  **(n=918)** | **Influenza**  **(n=24334)** | **Parainfluenza**  **(n=15268)** |
| --- | --- | --- | --- |
| **Severe complications** |  |  |  |
| Death cases | 2 (0.2%) | 11 (0.05%) | 4 (0.03%) |
| PICU admissions | 13 (1.4%) | 185 (0.8%) | 225 (1.5%) |
| Mechanical ventilation | 5 (0.5%) | 61 (0.3%) | 90 (0.6%) |
| Oxygen use | 7 (0.8%) | 91 (0.4%) | 204 (1.3%) |
|  |  |  |  |
| **Neurological complications** | 143 (15.5%) | 2373 (9.8%) | 1211 (7.9%) |
| All seizures | 139 (15.1%) | 2347 (9.6%) | 1204 (7.9%) |
| Febrile seizures | 133 (14.5%) | 2303 (9.5%) | 1142 (7.5%) |
| Seizures with fever | 2 (0.2%) | 8 (0.03%) | 9 (0.06%) |
| Breakthrough seizures with epilepsy | 4 (0.4%) | 36 (0.2%) | 53 (0.4%) |
| Encephalitis/encephalopathy | 4 (0.4%) | 37 (0.2%) | 14 (0.09%) |
|  |  |  |  |
| **Respiratory complications** | 65 (7.1%) | 1861 (7.7%) | 2672 (17.5%) |
| Croup | 61 (6.6%) | 585 (2.4%) | 884 (5.8%) |
| Pneumonia | 5 (0.5%) | 1290 (5.3%) | 1816 (11.9%) |

Data are n (%).

***Supplementary Table 2b:* Complications among the 3 viral infections between aged 6 to 11 years.**

|  | **SARS-CoV-2: Omicron BA.2**  **(n=226)** | **Influenza**  **(n=7878)** | **Parainfluenza**  **(n=1155)** |
| --- | --- | --- | --- |
| **Severe complications** |  |  |  |
| Death cases | 0 | 5 (0.1%) | 3 (0.3%) |
| PICU admissions | 8 (3.5%) | 69 (0.9%) | 45 (3.9%) |
| Mechanical ventilation | 3 (1.3%) | 21 (0.3%) | 16 (1.4%) |
| Oxygen use | 4 (1.8%) | 29 (0.3%) | 21 (1.8%) |
|  |  |  |  |
| **Neurological complications** | 28 (12.3%) | 334 (4.3%) | 47 (4.1%) |
| All seizures | 27 (11.9%) | 303 (3.9%) | 44 (3.8%) |
| Febrile seizures | 0 | 0 | 0 |
| Seizures with fever | 26 (11.5%) | 282 (3.6%) | 33 (2.9%) |
| Breakthrough seizures with epilepsy | 1 (0.4%) | 21 (0.3%) | 11 (1.0%) |
| Encephalitis/encephalopathy | 1 (0.4%) | 41 (0.5%) | 3 (0.3%) |
|  |  |  |  |
| **Respiratory complications** | 5 (2.2%) | 481 (6.1%) | 219 (19.0%) |
| Croup | 0 | 16 (0.2%) | 5 (0.4%) |
| Pneumonia | 5 (2.2%) | 466 (5.9%) | 214 (18.5%) |

Data are n (%).
