# Supplementary material for: Severity of SARS-CoV-2 Omicron BA.2 infection in unvaccinated hospitalized children: comparison to influenza and parainfluenza infections
Source: Emerg Microbes Infect. 2022 Jul 4;11(1):1742–50. doi: 10.1080/22221751.2022.2093135 (PMC9258055; doi:10.1080/22221751.2022.2093135)
Supplement: Supplemental Material [file TEMI_A_2093135_SM9542.zip › V8 Table S3.docx]

|  | **SARS-CoV-2 Omicron BA.2 vs Influenza** | | | | |  |  | **SARS-CoV-2 Omicron BA.2 vs Parainfluenza** | | | | |  | |  |
| --- | --- | --- | --- | --- | --- | --- | --- | --- | --- | --- | --- | --- | --- | --- | --- |
|  | 0-5 years old | |  | 6-11 years old | | Interaction |  | 0-5 years old | |  | 6-11 years old | | Interaction | | |
|  | Adjusted RR (95% CI) | p-value |  | Adjusted RR (95% CI) | p-value | p-value |  | Adjusted RR (95% CI) | p-value |  | Adjusted RR (95% CI) | p-value | | p-value | |
| **Severe complications** |  |  |  |  |  |  |  |  |  |  |  |  | |  | |
| Death cases | 3.4 (0.6-21.2) | 0.18 |  | 0 | 1† | NA |  | 10.4 (2.2-49.7) | 0.003** |  | 0 | 1 † | | NA | |
| PICU admissions | 1.5 (0.9-2.7) | 0.14 |  | 3.8 (1.8-7.9) | <0.001*** | 0.03* |  | 1.1 (0.6-1.9) | 0.74 |  | 1.2 (0.6-2.6) | 0.61 | | 0.32 | |
| Mechanical ventilation | 1.6 (0.7-4.1) | 0.29 |  | 4.5 (1.3-16.1) | 0.02* | 0.13 |  | 1.1 (0.4-2.7) | 0.85 |  | 1.3 (0.4-4.8) | 0.67 | | 0.48 | |
| Oxygen use | 1.7 (0.8-3.6) | 0.18 |  | 4.4 (1.5-12.9) | 0.006** | 0.08 |  | 0.6 (0.3-1.3) | 0.20 |  | 1.3 (0.4-3.8) | 0.67 | | 0.16 | |
| **Neurological complications** | 1.6 (1.4-1.9) | <0.001*** |  | 2.9 (2.0-4.3) | <0.001*** | 0.64 |  | 2.0 (1.7-2.3) | <0.001*** |  | 3.2 (2-5.1) | <0.001*** | | 0.02* | |
| All seizures | 1.6 (1.3-1.8) | <0.001*** |  | 3.1 (2.1-4.6) | <0.001*** | 0.04* |  | 1.9 (1.6-2.3) | <0.001*** |  | 3.1 (1.9-5.1) | <0.001*** | | 0.04* | |
| Encephalitis/ encephalopathy | 2.5 (0.8-7.7) | 0.10 |  | 0.8 (0.1-5.7) | 0.83 | 0.36 |  | 5.6 (1.3-23.9) | 0.02* |  | 4.5 (0.5-38.7) | 0.17 | | 0.86 | |
| **Respiratory complications** | 0.9 (0.7-1.2) | 0.49 |  | 0.4 (0.2-0.9) | 0.03* | 0.03* |  | 0.4 (0.3-0.5) | <0.001*** |  | 0.1 (0.1-0.3) | <0.001*** | | <0.001*** | |
| Croup | 2.0 (1.6-2.6) | <0.001*** |  | 0 | 1† | NA |  | 1.1 (0.8-1.4) | 0.55 |  | 0 | 1 † | | NA | |
| Pneumonia | 0.1 (0.02-0.3) | <0.001*** |  | 0.4 (0.2-0.9) | 0.03* | 0.08 |  | 0.05 (0.02-0.1) | <0.001*** |  | 0.1 (0.1-0.3) | <0.001*** | | 0.42 | |

***Supplementary Table 3:*** **Relative risks of complications for Omicron BA.2 in comparison to influenza and parainfluenza viral infections in different age groups.**

RR = relative risk, CI = confidence interval. Model was adjusted by age, sex and comorbidities. * p < 0.05, ** p < 0.01, *** p < 0.001.

† p-value of Fisher's Exact test is reported, in case one of the event has zero observation.
