# Supplementary material for: Severity of SARS-CoV-2 Omicron BA.2 infection in unvaccinated hospitalized children: comparison to influenza and parainfluenza infections
Source: Emerg Microbes Infect. 2022 Jul 4;11(1):1742–50. doi: 10.1080/22221751.2022.2093135 (PMC9258055; doi:10.1080/22221751.2022.2093135)
Supplement: Supplemental Material [file TEMI_A_2093135_SM9542.zip › V8 Table S4.docx]

***Supplementary Table 4.* Relative risks of complications for Omicron BA.2 cases in comparison to influenza during the influenza seasons in Hong Kong**

|  | **SARS-CoV-2 Omicron BA.2 vs influenza** | | | | |
| --- | --- | --- | --- | --- | --- |
|  | **During influenza seasons**^+^ | |  | **During the whole year** | |
|  | Adjusted  RR (95% CI) | p-value |  | Adjusted  RR(95% CI) | p-value |
| **Severe complications** |  |  |  |  |  |
| Death cases | 2.7 (0.3-22.2) | 0.36 |  | 2.7 (0.5-15.7) | 0.26 |
| PICU admissions | 2.0 (1.3-3.1) | 0.003** |  | 2.1 (1.3-3.3) | 0.001** |
| Mechanical ventilation | 2.2 (1.0-4.8) | 0.04* |  | 2.3 (1.1-4.9) | 0.03* |
| Oxygen use | 2.1 (1.1-3.9) | 0.02* |  | 2.3 (1.2-4.2) | 0.009** |
| **Neurological complications** | 1.6 (1.3-1.8) | <0.001*** |  | 1.6 (1.4-1.9) | <0.001*** |
| All seizures | 1.5 (1.3-1.8) | <0.001*** |  | 1.6 (1.4-1.9) | <0.001*** |
| Febrile seizures | 1.3 (1.1-1.5) | 0.001** |  | 1.4 (1.2-1.6) | <0.001*** |
| Seizures with fever | 2.7 (1.9-3.9) | <0.001*** |  | 3.0 (2.1-4.2) | <0.001*** |
| Encephalitis/ encephalopathy | 1.8 (0.8-4.3) | 0.19 |  | 1.8 (0.8-4.2) | 0.17 |
| **Respiratory complications** | 0.8 (0.7-1.0) | 0.09 |  | 0.8 (0.7-1.0) | 0.09 |
| Croup | 1.9 (1.5-2.5) | <0.001*** |  | 2.0 (1.5-2.6) | <0.001*** |
| Pneumonia | 0.2 (0.1-0.3) | <0.001*** |  | 0.2 (0.1-0.3) | <0.001*** |

RR = relative risk, CI = confidence interval. Model was adjusted by age, sex and comorbidities.

+ Influenza seasons in Hong Kong are January to April and July to August.

* p < 0.05, ** p < 0.01, ***, P<0.001.
