# Supplementary material for: Severity of SARS-CoV-2 Omicron BA.2 infection in unvaccinated hospitalized children: comparison to influenza and parainfluenza infections
Source: Emerg Microbes Infect. 2022 Jul 4;11(1):1742–50. doi: 10.1080/22221751.2022.2093135 (PMC9258055; doi:10.1080/22221751.2022.2093135)
Supplement: Supplemental Material [file TEMI_A_2093135_SM9542.zip › V8 Table S5.docx]

***Supplementary Table 5.* Clinical characteristics of hospital admissions of other SARS-CoV-2 variants (n=737).**

| Data period | 01/01/2020 – 01/11/2021 |
| --- | --- |
|  |  |
| Sex |  |
| Male | 401 (54.4%) |
| Female | 336 (45.6%) |
|  |  |
| Age mean in years | 5.7 (3.5) |
| 0 to 5 years | 400 (54.3%) |
| 6 to 11 years | 337 (45.7%) |
|  |  |
| Mortality and severe complications |  |
| Death cases | 0 |
| PICU admissions | 1 (0.1%) |
| Mechanical ventilation | 0 |
| Oxygen use | 0 |
|  |  |
| Neurological complications | 0 |
| All seizures | 0 |
| Febrile seizures | 0 |
| Seizures with fever | 0 |
| Breakthrough seizures with epilepsy | 0 |
| Encephalitis/encephalopathy | 0 |
|  |  |
| Respiratory complications | 8 (1.1%) |
| Croup | 2 (0.3%) |
| Pneumonia | 6 (0.8%) |
|  |  |

Data are n (%) unless otherwise specified.
